# Supplementary material for: Challenges with pediatric surgical financing and universal health coverage in Guatemala: A qualitative analysis
Source: PLOS Glob Public Health. 2022 Sep 21;2(9):e0000220. doi: 10.1371/journal.pgph.0000220 (PMC10021280; doi:10.1371/journal.pgph.0000220)
Supplement: S1 File — (PDF) [file pgph.0000220.s001.pdf]

Supplemental File 1: Clinician semi-structured in-depth interview guide

Challenges with pediatric surgical financing and universal health coverage in Guatemala: A qualitative analysis.

Introduction

The financing of pediatric surgical care remains challenging within public health and universal health coverage schemes, and there is an important knowledge gap due to lack of population level research on the financing of pediatric surgical care. This case study's objective is to evaluate the penetration of public health and universal health coverage schemes on the provision of pediatric surgical care. We are interviewing experts to better understand the financing of pediatric surgical care in Guatemala. Now, I have a few questions for an informal interview on this subject. Do I have your permission to record this informal interview that will be anonymous, confidential, and stored on a secure platform? All of the case study results will be shared with you.

Introducción

La financiación de la atención quirúrgica pediátrica permanece siendo un desafío dentro de los esquemas de la cobertura públicos y de la salud universal, y hay una falta de investigación de nivel poblacional sobre el financiamiento de la atención quirúrgica pediátrica y esto crea una brecha en conocimiento muy importante. Este estudio de caso tiene como objetivo evaluar la penetración de los esquemas de la cobertura públicos y de la salud universal en la provisión de atención quirúrgica pediátrica. Estamos entrevistando a expertos para aprender más de las finanzas de la atención quirúrgica pediátrica en Guatemala. Ahora, tengo unas preguntas para una entrevista informal de este sujeto. ¿Tengo su permisión grabar esta entrevista informal que va a ser anónimo, confidencial y guardado en una plataforma segura? Todos los resultados de este estudio de caso vamos a compartir con usted.

---

1) In your public health system, where do you think that the greatest inequalities in financing for pediatric surgical coverage exist?

> ¿En su sistema de salud pública, donde usted cree que existan las desigualdades más grandes en la cobertura financiera de la atención quirúrgica pediátrica?

2) What are primary barriers to sufficient financing of pediatric surgery in the Guatemalan public health system?

> ¿Cuales son los desafíos más grandes en el financiamiento de la atención quirúrgica pediátrica en el sistema de salud pública en Guatemala?

3) Where does the infrastructure for pediatric surgery, or pediatric healthcare in general, work? Where does it not? (i.e. human resources for health, governance, health finance, service delivery, health information systems, medical products & supply chain)

¿Dónde funciona la infraestructura para la cirugía pediátrica, o el cuidado médico pediátrico en general? ¿Dónde no funciona? (Por ejemplo, los recursos humanos para la salud, la gobernanza, las finanzas de salud, prestación de servicio, productos médicos y cadena de suministro)

4) How much of pediatric surgical care is covered by public health insurance schemes and how much is purchased by household funds (estimate as a percent)?

¿Cuánto de la atención quirúrgica pediátrica es comprado por esquemas de cobertura publicas y cuanto por fondos domésticos o del bolsillo (en una estimación de un porcentaje)?

5) Who are the primary beneficiaries of pediatric surgical care coverage in the public health system? Does this include civil servants, private sector employees, members of differing socioeconomic levels or targeted age groups?

¿Quiénes son los beneficiarios primarios de la atención quirúrgica pediátrica en el sistema de salud pública? ¿Incluye este grupo a funcionarios (o sea servidores públicos), empleados del sector privado, miembros de niveles socioeconómicos diferentes o un grupo de edad especifico?

6) Who pays for these pediatric surgical procedures in the public health system, or who is the primary financing agent of pediatric surgery in the country? (i.e. local government, central government, social security contributions, NGO contracts, etc.).

¿Quién paga para estos procedimientos quirúrgicos pediátricos en el sistema de salud pública, o quien es el agente primario del financiamiento de la atención quirúrgica pediátrica en el país? (por ejemplo, el gobierno local, el gobierno central, contribuciones de seguridad social, contractos de ONG, etc.)?

Clinician Specific Questions:

---

1) Do the IGSS, MSPAS, and Armed Forces Health System benefit packages (i.e. public health system benefits packages) include pediatric surgical coverage?

¿Estos esquemas de cobertura publicas de IGSS (el Instituto Guatemalteco de Seguridad Social), MSPAS (el Ministerio de Salud Pública) y el Sanidad Militar incluyen la atención quirúrgica pediátrica?

2) What is the estimated out-of-pocket cost for inguinal hernia repair, open fracture fixation, gastroschisis repair and male circumcision?

¿Cuál es el gasto extra (o sea del bolsillo) estimado para reparación de hernia inguinal, fijación de fractura abierta, reparación de gastrosquisis y circuncisión masculina?

3) What are the points of contact with the health system for a child that requires pediatric surgical care in the public health system (i.e. must they first pay to visit a primary level facility, then pay to access a more specialized facility, etc.)?

¿Qué son los puntos de contacto con el sistema de salud para un niño o una niña que requiera el cuidado quirúrgico en el sistema público de salud (por ejemplo, tenga que pagar para visitar a un hospital del primer nivel, luego tenga que pagar para acceder a un hospital con más especialidad, etc.)?

4) Do pediatric surgical procedures require user-fees, copayments or cost-sharing by the user? If yes, can you estimate the percentage of the total cost of pediatric surgical procedures that these fees comprise?

¿Requieren los procedimientos quirúrgicas pediátricas cuotas de usuarios, copago o reparto de costos por la persona que los usan? ¿Si las requieren, puede usted estimar al porcentaje del costo total de los procedimientos quirúrgicos pediátricos que es de estas cuotas?

5) Can you describe any informal fees (i.e. medications, supplies, etc.) that the patient must pay to access pediatric surgical care? How often do these fees exist?

¿Puede usted describir a los pagos informales (por ejemplo, los medicamentos, provisiones, etc.) que el paciente tiene que pagar para acceder la atención de la quirúrgica pediátrica? ¿Si existan, qué tan frecuentes son estos pagos informales?

6) How often, for example in the last month, have patients had to pay for or bring medical supplies for surgery?

¿Con cuanta frecuencia, por ejemplo en el último mes, los pacientes han tenido que pagar para los suministros médicos para la cirugía?

How many of these patients return for surgery?

¿Cuántos de estos pacientes regresan para su cirugía?

How many of these patients do not return for surgery?

¿Cuántos de estos pacientes no regresan para su cirugía?

How many patients return much later with further complications?

¿Cuántos de estos pacientes regresan mucho después con más complicaciones?

7) How does stockout of surgical supplies affect patient fees for those requiring pediatric surgical procedures?

¿Cómo se afecta la falta de suministros quirúrgicos las tarifas que pagan los pacientes que requieren la cirugía pediátrica?

8) Briefly describe the public/private mix of pediatric surgical care financing in the public health system.

¿Puede usted describir la mezcla de financiamiento de la atención quirúrgica pediátrica entre el sector privado y el sector público?

9) How frequently do you believe families are forced into impoverishing or catastrophic health expenditure to pay for pediatric surgical procedures?

¿Con cuánto frecuencia cree usted que las familias son arrojadas o forzadas en la pobreza, o empobrecidos, por pagar para la atención quirúrgica pediátrica?

10) How does lack of access to financing for health infrastructure and supplies, especially in rural areas, affect pediatric surgical coverage (ex. frequent medicine and supply stockouts, etc.)?

¿Cómo afecta la falta del acceso a la financiación para la infraestructura de salud y las provisiones, especialmente en áreas rurales, la cobertura de la atención quirúrgica pediátrica (por ejemplo, falta de medicina y provisiones)?

11) In regard to IGSS, many patients purchase additional insurance coverage. Do patients often purchase additional insurance coverage for pediatric surgery? Please explain.

Con relación a IGSS, muchos pacientes compran cobertura de salud adicional a lo que les da este programa. ¿Compran pacientes frecuentemente cobertura de salud adicional para la cirugía pediátrica? ¿Por favor, puede usted explicar más?

12) Please describe the criteria and motivations for inclusion of pediatric surgical, or medical procedures in general, in the benefit package of public insurance schemes (ex. cost effectiveness, affordability, financial protection, political context etc.).

¿Por favor, puede describir los criterios de la inclusión de la atención quirúrgica pediátrica, o cualquier forma del cuidado médico en general, en el paquete de beneficios bajo de los esquemas de la cobertura de salud en el país (por ejemplo, que tan rentables son, asequibilidad, protección financiera, el contexto político etc.)?

13) What stakeholders run the public pediatric surgical and pediatric healthcare plan? (e.g. who decides how much financing this specialty gets and what services are included?)

¿Quiénes son los depositarios que operan el plan de cobertura pública de la atención quirúrgica pediátrica o lo del cuidado médico pediátrico más en general? (¿o sea, quien decide el presupuesto de esta especialidad y cuantos servicios van a ser incluidos en el paquete de beneficios?)

14) Does pediatric surgical care in the public health system have earmarked funding from a central government source? If so, please describe.

> ¿Tiene la cobertura de la quirúrgica pediátrica en el sistema de salud pública fondos destinados de una fuente del gobierno central?

15) Was the initial budget for pediatric surgery in the benefits package defined based on actuarial studies? Guesswork? Examples from previous programs? An amount the government was willing to spend?

> ¿Sería el presupuesto original para la atención quirúrgica pediátrica en el paquete de beneficios basado en estudios actuariales, conjeturas, programas anteriores, la cantidad que el gobierno estaba dispuesto a gastar?

16) What were the motivations, precursors, issues, domestic and political context, donor roles in the country when pediatric care was included in the benefits package?

> ¿Cuáles fueron las motivaciones, precursores, problemas, contextos domésticos y políticos, y roles de donantes en el país cuando la atención de el cuidado medico pediátrico fue incluido en el paquete de beneficios?

17) One of the noted health systems challenges is the change of administrations every 4 years, often including high-level health sector officials. How does this affect hospital and patient fees? (e.g. hospital fees on a sliding scale before Colom was president, no longer exist now; e.g. changes in if there is a fixed budget for pediatric surgical care, etc.)

> Uno de los desafíos del sistema de salud que se observa es el cambio de administraciones cada cuatro años, a menudo incluyendo los oficiales de salud de alto nivel. ¿Cómo afecta esto a las cuotas del usuario o del paciente? (Por ejemplo, había cuotas de usuario para acceder a la atención quirúrgica basadas en el estatus socioeconómico del paciente antes de El Presidente Colom que ya no existen ahora; Por ejemplo, cambios en si hay un presupuesto fijado para la atención quirúrgica pediátrica, etc.)
