# Supplementary material for: Challenges with pediatric surgical financing and universal health coverage in Guatemala: A qualitative analysis
Source: PLOS Glob Public Health. 2022 Sep 21;2(9):e0000220. doi: 10.1371/journal.pgph.0000220 (PMC10021280; doi:10.1371/journal.pgph.0000220)
Supplement: S2 File — Developed from: Tong A, Sainsbury P, Craig J. Consolidated criteria for reporting qualitative research (COREQ): A 32-item checklist for interviews and focus groups. International Journal for Quality in Health Care. 2007. Volume 19, Number 6: pp. 349–357. (PDF) [file pgph.0000220.s002.pdf]

**Supplemental File 2**

**COREQ (COnsolidated criteria for REporting Qualitative research) Checklist**
**Pediatric surgical financing and universal health coverage in Guatemala: A qualitative assessment of financing policy and practice**

| Domain | Item Number | Comment | Reported on Page Number or N/A |
|---|---|---|---|
| **Domain 1: Research Team and Flexibility** | | | |
| *Personal Characteristics* | | | |
| Interviewer/Facilitator | 1 | The lead author KL conducted the individual interviews in Spanish. | 9 |
| Credentials | 2 | All authors and research assistants have extensive experience in conducting qualitative health research and completed study training. Language credentials are as follows: Interviewer KLR is fluent in Spanish-speaking and trained in medical Spanish translation. | N/A |
| Occupation | 3 | KL was a graduate student working with researchers in Guatemala. HR is a pediatric surgeon with extensive research experience in Guatemala. BH was a research coordinator at Duke with extensive research experience in Guatemala. | N/A |
| Gender | 4 | Two of the researchers conducting this work were female. One was male. | N/A |
| Experience and Training | 5 | All research team members have training in conducting qualitative health research. Qualitative work was also guided by researchers with extensive qualitative research at Duke Global Health Institute, namely Dr. Melissa Watt. | N/A |
| *Relationship with Participants* | | | |
| Relationship established | 6 | Interviewer (KL) did not have any affiliations or relationship with participants in this study to ensure that the collection of data was unbiased. | N/A |
| Participant knowledge of the interviewer | 7 | Each participant was introduced to the researcher and the study team at the beginning of each interview. The participant was | N/A |

| | | | |
|---|---|---|---|
| | | informed of how the data would be used and the purpose of the researcher and the study. | |
| *Interviewer characteristics* | 8 | Interviewer was bilingual English and Spanish speaking. | 9 |
| ***Domain 2: Study Design*** | | | |
| *Theoretical framework* | | | |
| Methodological orientation and Theory | 9 | The World Bank Group's Universal Health Coverage Study Series (UNICO) and World Health Organization's health systems building block framework were used to lead the development of the interview guide and codebook. A thematic analysis was conducted. | 9 |
| Participant selection | | | |
| Sampling | 10 | Participants were selected using purposive sampling methodology. | 8 |
| Method of approach | 11 | Key stakeholders were identified and solicited to participate in the study through local content expert messaging. Those selected to participate were also asked to pass the information on to stakeholders (snowball sampling). | N/A |
| Sample Size | 12 | Qualitative interviews (n=20) | |
| Non-participation | 13 | No approached participant refused to participate in the study. | N/A |
| Setting | | | |
| Setting of data collection | 14 | Individual interviews were conducted privately in an office or the clinical unit. | 9 |
| Presence of nonparticipants | 15 | Researchers and participants were the only people present at the time of the interview. | N/A |
| Description of sample | 16 | Sample characteristics from SSIs are reported in the methods section and presented in Table 1. | 8 |
| *Data Collection* | | | |
| Interview guide | 17 | The interview guide was developed using The World Bank Group's Universal Health Coverage Study Series (UNICO) and World Health Organization's health systems building block framework. Interview guide was | 9 |

| | | | |
|---|---|---|---|
| | | reviewed for language and cultural appropriateness. | |
| Repeat interviews | 18 | No repeat interviews were conducted. | N/A |
| Audio/visual recording | 19 | All interviews were audio recorded on a password protected device. Audio recording were deleted after uploading to secure study folder. | 10 |
| Field notes | 20 | The interviewer wrote field notes during interviews depending on the participant. | N/A |
| Duration | 21 | Each interview varied in duration, but average length was 27 minutes (range 00:12:07-01:27:22). | 9 |
| Data saturation | 22 | Data saturation was achieved approximately at 20 interviews and was defined by no new themes discussed by participants. | 26 |
| Transcripts returned | 23 | No transcripts were returned to participants. | N/A |
| **Domain 3: Analysis and Findings** | | | |
| *Data Analysis* | | | |
| Number of data coders | 24 | Each transcript was coded by KL. Twenty-five percent were coded independently by two coders (KL and BH) for interrater reliability and codebook assessment. Memos were reviewed by KL, BH, and HR. | 10 |
| Description of the coding tree | 25 | Codes and definitions were established a priori and derived from the interview guide and thorough literature review. | 10 |
| Derivation of themes | 26 | Themes were defined from the interview guide, thorough literature review, and emergence of themes among participants. | 10 |
| Software | 27 | NVivo version 12 was used to organize data analysis. | 10 |
| Participant checking | 28 | Participant checking was not conducted. | N/A |
| Reporting | | | |
| Quotations presented | 29 | Quotations were selected to represent each identified theme in the results. Participants were kept anonymous and each quotation is attributed to “#, role”. | N/A |

| | | | |
|---|---|---|---|
| Data and findings consistent | 30 | There is consistency between data presented and findings. | N/A |
| Clarity of major themes | 31 | Yes, major themes are clearly identified. | N/A |
| Clarity of minor themes | 32 | Yes, minor themes are clearly identified. | N/A |

Developed from: Tong A, Sainsbury P, Craig J. Consolidated criteria for reporting qualitative research (COREQ): a 32-item checklist for interviews and focus groups. International Journal for Quality in Health Care. 2007. Volume 19, Number 6: pp. 349 – 357
