# Supplementary material for: Challenges with pediatric surgical financing and universal health coverage in Guatemala: A qualitative analysis
Source: PLOS Glob Public Health. 2022 Sep 21;2(9):e0000220. doi: 10.1371/journal.pgph.0000220 (PMC10021280; doi:10.1371/journal.pgph.0000220)
Supplement: Attachment — Submitted filename: Comments_to_reviewers_2.pdf [file pgph.0000220.s003.pdf]

4 August 2022

RE: "Challenges with pediatric surgical financing and universal health coverage in Guatemala: A qualitative analysis"

Dear Reviewer #2,

Thank you very much for your thoughtful comments and suggestions regarding our manuscript, "Challenges with pediatric surgical financing and universal health coverage in Guatemala: A qualitative analysis". We appreciate your review and find these comments helpful in revising this manuscript. Please find our responses to your comments below and the corresponding edits in the attached manuscript with tracked changes.

Review of "Challenges with pediatric surgical financing and universal health coverage in Guatemala: A qualitative analysis"

Comments:

The topic of the paper is well-motivated in the introduction.

I believe that the introduction section is too short, and it does not include several points of the investigation, which would be interesting to read in the first section of the paper. For example, a summary of the context of the research for Guatemala, the methods, and the main findings may be presented in the introduction.

> **Thank you for your comments regarding the “Introduction” section. We have expanded our introduction to include more background information on the Guatemalan context and a more thorough introduction to the study’s methods in both this revision and the prior revision. We introduce key financing issues in Guatemala in the “Introduction” section and our study’s key findings in the results and discussion sections.**

The “Methods” section is too short.

The “Setting” section, or a part of it, may be included in the Introduction to describe the context of the research for Guatemala.

There are some short sections and the material presented in those sections is for some cases belongs to the introduction and for some cases it belongs to a methods section. Please review the organization of the different sections of the paper. Nevertheless, in the separate sections, I believe that the detail of the method is well-presented. Please put them together into a longer methods section.

> **Thank you for your suggestions for the “Methods” section. We have elaborated on the Guatemalan context in the “Introduction” section and have included information from the “Setting” section as recommended. We have also reorganized the “Methods” subsections according to your suggestions. The section is now organized as 1) Study setting and subject recruitment, 2) Key informant interview procedures, 3) Applied thematic analysis, and 4) Ethical approval and hope that the new organization and length of the section adequately addresses your comments.**

I believe that a possible structure of the paper could be: 1) Introduction; 2) Methods; 3) Results; 4) Discussion; 5) Conclusions.

The results, discussion, and conclusion sections are well-presented.

> **Thank you for your encouraging comments on these sections.**

Sincerely,

Kelsey R. Landrum
